# Keratinocyte-derived circulating microRNAs in extracellular vesicles: a novel biomarker of psoriasis severity and potential therapeutic target

**DOI:** 10.1186/s12967-024-05030-z

**Published:** 2024-03-04

**Authors:** Young Joon Park, Dong Chan Kim, Soo-Jin Lee, Han Seul Kim, Ji Young Pak, Junho Kim, Jae Youn Cheong, Eun-So Lee

**Affiliations:** 1 Department of Dermatology, Ajou University School of Medicine, Ajou University Hospital, 164, World Cup-Ro, Yeongtong-Gu, Suwon-Si, Gyeonggi-Do 16499 South Korea; 2 Department of Biomedical Sciences, Graduate School of Ajou University, Suwon, Korea; 3 Ajou Translational Omics Center, Ajou University Medical Center, Suwon, Korea

**Keywords:** Biomarkers, Extracellular vesicles, Keratinocytes, microRNAs, miR-625-3p, Psoriasis

## Abstract

**Background:**

Psoriasis is a chronic inflammatory disorder characterized by pathogenic hyperproliferation of keratinocytes and immune dysregulation. Currently, objective evaluation tools reflecting the severity of psoriasis are insufficient. MicroRNAs in extracellular vesicles (EV miRNAs) have been shown to be potential biomarkers for various inflammatory diseases. Our objective was to investigate the possibility of plasma-derived EV miRNAs as a marker for the psoriasis disease severity.

**Methods:**

EVs were extracted from the plasma of 63 patients with psoriasis and 12 with Behçet’s disease. We performed next-generation sequencing of the plasma-derived EV miRNAs from the psoriasis patients. Real-time quantitative reverse transcription polymerase chain reaction (qRT-PCR) was used to validate the level of EV miRNA expression. In situ hybridization was used to discern the anatomical location of miRNAs. qRT-PCR, western blotting, and cell counting kits (CCKs) were used to investigate IGF-1 signaling in cells transfected with miRNA mimics.

**Results:**

We identified 19 differentially expressed EV miRNAs and validated the top three up-and down-regulated EV miRNAs. Among these, miR-625-3p was significantly increased in patients with severe psoriasis in both plasma and skin and most accurately distinguished moderate-to-severe psoriasis from mild-to-moderate psoriasis. It was produced and secreted by keratinocytes upon stimulation. We also observed a significant intensification of IGF-1 signalling and increased cell numbers in the miR-625-3p mimic transfected cells.

**Conclusions:**

We propose keratinocyte-derived EV miR-625-3p as a novel and reliable biomarker for estimating the severity of psoriasis. This biomarker could objectively evaluate the severity of psoriasis in the clinical setting and might serve as a potential therapeutic target.

*Trial registration* None.

**Supplementary Information:**

The online version contains supplementary material available at 10.1186/s12967-024-05030-z.

## Background

Psoriasis is a relatively common chronic inflammatory disease characterized by pathogenic hyperproliferation of keratinocytes and immune dysregulation [1]. Despite the evolution of disease treatment, tools for evaluating disease activity are still lacking. The only currently widely used clinical subjective assessment tools to evaluate psoriasis are the Psoriasis Area and Severity Index (PASI) and body surface area (BSA) [2]. Unfortunately, these are subjective evaluation tools that show relatively high inter-rater variability [3]. Moreover, the recent coronavirus disease 2019 breakout has underscored the need for patients to receive clinical services remotely [4]. Thus, the need for objective markers that reflect the severity of psoriasis has emerged. Candidate biomarkers including cytokine, chemokines and adipokines have been studied exhaustively, yet none have become part of current routine practice. [5]

Extracellular vesicles (EVs) are small structures composed of a phospholipid bilayer derived from the cell membrane. Specifically, EVs are classified into exosomes (30–150 nm), micro-vesicles (100–1500 nm), and apoptotic bodies (500–2000 nm), depending on their size and biogenesis pathway [6]. If EVs are released into the extracellular space, they can facilitate communication between cells by transporting their bioactive contents, which include nucleic acids, proteins, and various microRNAs (miRNAs), to the recipient cells [6–8]. EV miRNAs are found in different biofluids, cells, and tissues [7]. These EV miRNAs have demonstrated potential as diagnostic markers of various inflammatory diseases including psoriasis. [9–11]

Given the noticeable scarcity of studies focusing on objective markers for psoriasis severity, particularly those utilizing EV miRNAs, our objective was to identify a reliable biomarker of psoriasis disease activity using plasma-derived EV miRNAs. We analyzed the levels of EV miRNAs in both the plasma and skin of patients and identified keratinocytes as the origin of differentially expressed miRNA (DE miRNA) in the EVs. Furthermore, we showed that the keratinocyte-derived miRNA promotes keratinocyte proliferation, a hallmark of psoriasis.

## Methods

### Study subjects, acquisition of biospecimens, and study flow

This study included patients diagnosed with psoriasis between May 2016 and March 2021, and patients diagnosed with Behçet’s disease (BD) between April 2016 and May 2020. The diagnosis of psoriasis was made based on clinical characteristics and histological features on skin biopsy. None of the patients with psoriasis received systemic treatment or phototherapy at the time of sample collection. PASI and BSA were also evaluated on the day of sampling. Patients with BD met the International Criteria for BD [12] and the Japanese Criteria for BD [13]. BD patients were chosen as a control group for our research, given their shared autoinflammatory traits and T-helper 1 and T-helper 17 dominant immune response [1, 13]. Plasma samples were collected from 63 patients with psoriasis (8 patients for screening, 42 for validation, and 20 for pre- and post-treatment comparisons; some patients participated in screening, validation, and pre-and post-treatment comparisons) and 12 patients with BD.

The blood samples for obtaining the patient's plasma were collected in an EDTA-containing tube and immediately centrifuged at 2000 × g for 10 min at 4 °C to remove cellular components. The plasma samples were divided into aliquots in microcentrifuge tubes and stored at −80 ℃ in a deep freezer until used for experimental analysis. Tissue samples from psoriatic lesions were obtained using a 3-mm disposable biopsy punch, and snap-frozen tissue samples from patients were stored in a nitrogen tank at the Ajou University Hospital Human Biobank until analysis. Using plasma samples from eight patients (five patients with PASI ≥ 10 and three with PASI < 5), we performed next-generation sequencing (NGS) of miRNAs to screen DE miRNAs. Forty patients were further enrolled to validate the screened DE miRNAs (two patients participated in both screening and validation). We performed real-time quantitative reverse transcription-polymerase chain reaction (qRT-PCR) to validate the DE miRNAs identified by screening DE miRNAs using NGS. Skin samples were obtained from 18 patients who consented to additional tissue collection at the time of the biopsy. The tissue samples were homogenized in QIAzol lysis Reagnet (Qiagen, Hilden, Germamy) using TissueLyser II (Qiagen, Hilden, Germamy). Total RNA was extracted using the TRIzol-based extraction method.

### Isolation of EVs, RNA extraction, and NGS of miRNAs

After thawing the plasma, it was centrifuged for 10 min at 300 × g and 4 ℃, followed by centrifugation at 10,000 × g for 30 min and 4 ℃ for 30 min. From the supernatants, EVs were extracted using the miRCURY exosome isolation kit (Qiagen, Hilden, Germany) and total miRNA was extracted from the exosomes using a miRNA isolation kit (miRNeasy Serum/Plasma Kit; Qiagen, Hilden, Germany) according to the manufacturer's protocols. The total RNA quantity and quality were assessed by spectrophotometry (NanodropTM ND-1000, Thermo Fisher Scientific, Copenhagen, Denmark). miRNA sequencing was performed by Theragen Bio Co. Ltd. (Suwon, South Korea). Please see “Supplementary Materials and Methods” for a detailed description of the process.

### Size exclusion chromatography and ultracentrifugation

As described previously, EVs from the selected plasma samples of psoriasis patients were isolated using mini-size exclusion chromatography (mini-SEC) [14]. Briefly, plasma was centrifuged for 10 min at 300 × g and 4 ℃, followed by centrifugation at 10,000 × g for 30 min, 4 ℃ for 30 min. The supernatant obtained after the second centrifugation was loaded on the Sepharose CL-2B (Sigma Aldrich, St. Louis, MO, USA) column. The flow-through was collected in 6 fractions of 1 ml each. The fractions containing EVs (fractions #4, #5, and #6) were pooled and transferred to a new ultracentrifuge tube and then ultracentrifuged at 100,000 × g for 70 min using an SW32 Ti rotor (Beckman Coulter, California, USA) to pellet EVs. The pellets of EVs were resuspended in TRIzol reagent (Thermo Fisher Scientific, Copenhagen, Denmark) for RNA isolation in phosphate-buffered saline (PBS) for transmission electron microscopy and nanoparticle tracking analysis. Additional details regarding experimental materials and methods can be found in the “Supplementary Materials and Methods” section.

### Statistical analysis

All data are presented as mean ± standard deviation. Statistical analyses were performed using GraphPad Prism 9.3.1 (GraphPad Software Inc., La Jolla, CA, USA) and the R statistical program 4.2.1 (R Foundation for Statistical Research, Vienna, Austria). When comparing two groups, we used an unpaired Student's t-test, applying Welch's correction when an F-test indicated differing variances between the sample groups. A paired t-test was used to compare values of miRNA levels between pre-treatment and post-treatment samples. Spearman's correlation test was used to assess the relationship between the validated miRNAs and their association with PASI and BSA scores. For multiple comparisons, we employed an unpaired one-way analysis of variance (ANOVA). Statistical significance was established at P < 0.05.

## Results

### Hsa-miR-625-3p expression is significantly increased in both the plasma and skin of patients with psoriasis and is associated with PASI and BSA

**Fig. 1:**
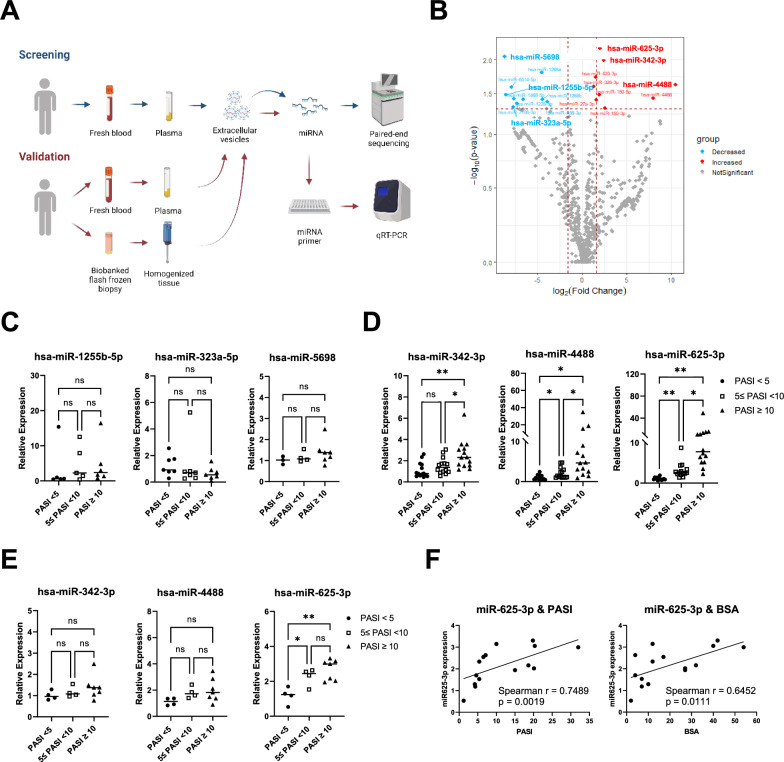
Differentially expressed (DE) miRNAs in extracellular vesicles (EV miRNAs) identified from blood and skin of patients with severe and mild psoriasis. **A** Experimental scheme for acquisition and analysis of EV miRNA (Screening phase shown in blue arrows and validation phase shown in red arrows). **B** DE miRNAs on volcano plot (Selected EV miRNAs shown in bold). Blue and red colored dots indicate miRNAs with |log2 fold change|≥ 2 and *P* ≤ 0.05, and grey colored dots represent miRNAs with no significant changes. **C** and **D** qRT-PCR results of selected **C** downregulated and **D** upregulated miRNAs in the plasma of psoriasis patients. **E** qRT-PCR results of upregulated miRNAs in skin of psoriasis patients. For comparisons of differences between groups **C**-**E**, ordinary one-way ANOVA was used. (**F**) The skin miR-625-3p level plotted against PASI and BSA. EV miRNAs; miRNAs in extracellular vesicles. The significance of the correlation was assessed by the Spearman's rank correlation test. *P < 0.05 and **P < 0.01

The baseline demographics of patients with psoriasis are summarised in Table 1. The EV miRNAs extracted from 8 patients (clinical characteristics described in Table 1 and characteristics of isolated EVs shown in Additional file 1: Figure S1) underwent NGS to identify candidate miRNAs (Fig. 1A, blue arrows). Out of the 805 miRNAs detected, we identified 19 DE miRNAs, including 9 upregulated and 10 downregulated miRNAs (Fig. 1B and Additional file 1: Table S1). We carefully chose three DE miRNA candidates based on their fold change and p-value: miR-625-3p, miR-4488, and miR-342-3p from the upregulated miRNAs, and miR-5698, miR-1255b-5p, and miR323a-5p from the downregulated miRNAs. To validate possible miRNAs for use as biomarkers, we performed qRT-PCR of the selected miRNAs with a larger group of patients (Fig. 1A, red arrows), classified by their PASI score (PASI < 5, 5 ≤ PASI < 10, and PASI ≥ 10, with each group consisting of 14 patients). Although the selected downregulated miRNAs did not display significant expression differences between the groups (Fig. 1C), all the chosen upregulated miRNAs exhibited significant increases between the PASI < 5 and the PASI ≥ 10 groups. Furthermore, both miR-4488 and miR-625-3p showed significant differences between the PASI < 5 and 5 ≤ PASI < 10 groups (Fig. 1D). In particular, miR-625-3p showed a significant difference between the PASI < 5 and 5 ≤ PASI < 10 groups, as well as between the PASI < 5 and PASI ≥ 10 groups using EV miRNAs isolated from the skin (Fig. 1E). miR-625-3p was the only EV miRNA from the skin with a strong association (ρ ≥ 0.60) [24] between both PASI and BSA (Fig. 1F), whereas other miRNAs demonstrated limited associations. (miR-4488 and PASI: Spearman’s ρ = 0.5862, p = 0.0236; miR-342-3p and PASI: Spearman’s ρ = 0.3718, p = 0.1719; Additional file 1: Figure S2).

**Table 1:** Demographic and clinical characteristics of the patients with psoriasis

Patient numbers	3	5	*p*-value	14	14	14	*p*-value	10	10	*p*-value
Characteristics	Screening			Validation				Treatment response		
	PASI < 5	10 ≥ PASI		0 < PASI < 5	5 $\le$ PASI < 10	10 ≥ PASI		Poor responders*	Good responders*	
Age (mean ± SD, yr)	36.3 ± 5.6	52.6 ± 15.4	0.1124	36.3 ± 9.3	40.1 ± 13.4	37.1 ± 11.6	0.6837	39.8 ± 15.8	41.7 ± 15.5	0.7890
Sex (M:F)	2:1	4:1		7:7	10:4	8:6		7:3	7:3	
BMI (mean ± SD, kg/m^2^ or lb/ inches $\times$ 703)	24.2 ± 1.1	25.5 ± 1.0	0.2032	25.5 ± 4.2	24.1 ± 3.3	26.4 ± 5.1	0.3755	26.5 ± 3.2	25.4 ± 2.4	0.3888
Initial PASI (mean ± SD)	3.9 ± 0.5	13.6 ± 1.9	0.0002	3.5 ± 1.0	6.5 ± 1.0	18.9 ± 6.0	< 0.0001	10.8 ± 5.1	13.1 ± 5.7	0.3251
Initial BSA (mean ± SD, %)	4.7 ± 2.1	23.4 ± 4.0	0.0003	6.1 ± 4.2	9.4 ± 5.3	31.9 ± 12.3	< 0.0001	15.7 ± 16.1	20.6 ± 11.5	0.4457
Disease duration (yr)	6.3 ± 3.3	12.8 ± 8.9	0.2486	10.5 ± 8.8	10.0 ± 8.3	12.5 ± 9.8	0.7575	10.8 ± 5.9	13.8 ± 5.2	0.2430
Psoriasis onset age(mean ± SD, yr)	30.0 ± 2.4	39.8 ± 11.8	0.1749	25.8 ± 7.3	30.1 ± 11.8	24.7 ± 13.8	0.4484	29.0 ± 16.3	27.9 ± 16.3	0.8816
Joint involvement (%)	0	0	> 0.9999	0	0	14.2	0.1225	0	0	> 0.9999
Nail involvement (%)	66.7	80	> 0.9999	21.4	14.2	42.9	0.2017	50	40	> 0.9999

SD, standard deviation; BMI, body mass index; PASI, Psoriasis Area and Severity Index; BSA, body surface area. All participants in the study were of Asian ethnicity. 'Good responders' were defined as patients who achieved a PASI 50 response, while 'poor responders' were those who did not achieve a PASI 50 response following treatment. To assess the differences between the groups, Welch’s t-test was employed for the screening phase and for the evaluation of treatment response. One-way ANOVA was utilized in the validation phase. For comparisons of joint and nail involvement, Fisher’s exact test was used for the screening phase and for evaluating treatment response, while the Chi-square test was applied in the validation phase

### EV miR-625-3p most accurately differentiates mild and moderate-to-severe psoriasis, is psoriasis-specific, and reflects treatment response.

**Fig. 2:**
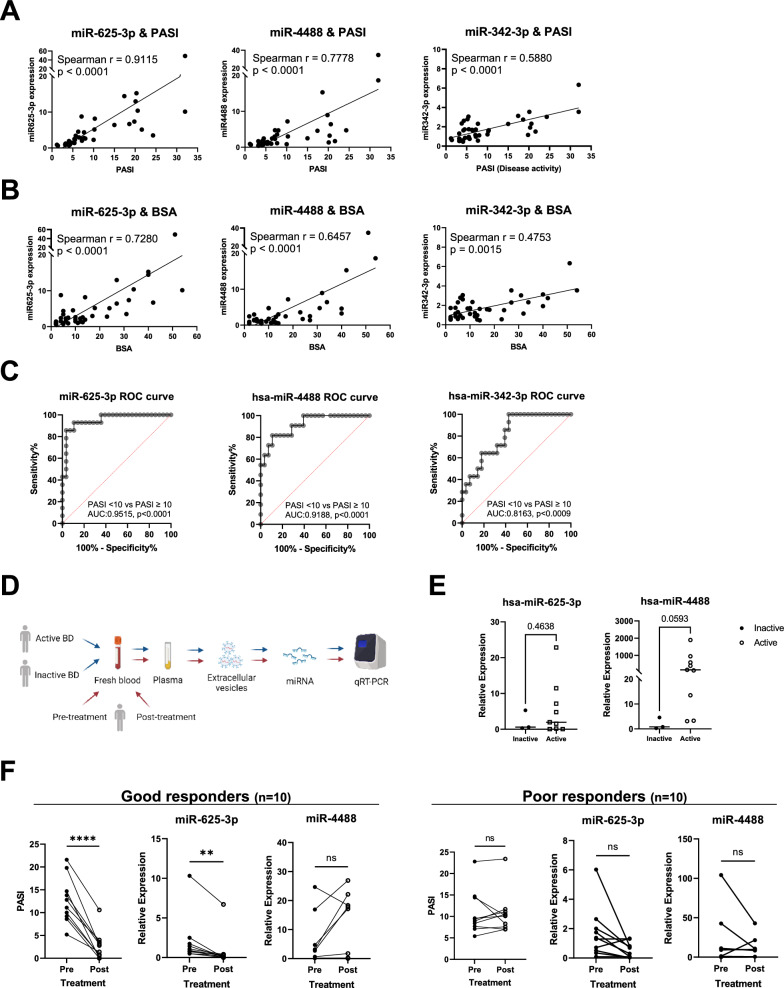
EV miR-625-3p is highly associated with PASI and BSA, most accurately differentiates mild and moderate-to-severe psoriasis, is psoriasis-specific, and declines significantly when successfully treated. **A** and **B** Plasma miR-625-3p, miR-4488 and miR-342-3p level plotted against **A** PASI and **B** BSA. The significance of the correlation was tested using the Spearman's rank correlation test. **C** Receiver Operating Characteristic (ROC) curves and associated AUC value of each upregulated EV miRNAs. A higher area under the ROC curve (AUC) indicates superior model discrimination, ranging from 0 (none) to 1 (perfect). **D** Experimental scheme for analysis of EV miRNA level in BD patients and before-and-after treatment. **E** qRT-PCR results of plasma levels of miR-625-3p and miR-4488 according to disease activity of patients with BD. The difference between groups was assessed using ordinary one-way ANOVA. **F** Expression level of EV miRNAs from plasma of patients before (Pre) and after (Post) treatment. Paired t-test was used to compare the two groups. *P < 0.05

We also confirmed a significant association between EV miR-625-3p and PASI (Fig. 2A). All selected miRNAs showed a significant correlation (p < 0.0001) between their expression levels and PASI, but miR-625-3p showed an exceptionally high level of correlation. A similar result was observed in its correlation with BSA; it had the highest correlation coefficient value compared to other miRNAs (Fig. 2B). In the receiver operative characteristic (ROC) curve, the area under the curve (AUC) value was also the highest for miR-625-3p, with an exceptionally high value of 0.9515, thereby confirming its diagnostic value as a biomarker for differentiating mild-to-moderate psoriasis (PASI < 10) from moderate-to-severe psoriasis (PASI ≥ 10) (Fig. 2C). Based on these results, we narrowed the potential biomarkers to miR-625-3p and miR-4488. To exclude the possibility that the miRNAs are markers of inflammation, which is not psoriasis-specific, we analyzed the EV miRNAs of BD patients (Fig. 2D, blue arrows). When comparing patients with active BD and those with inactive BD, albeit statistically insignificant (p = 0.0593), we observed an increasing tendency in miR-4488 in symptomatic BD patients (Fig. 2E). The results suggest that miR-4488, in contrast to miR-625-3p, is likely not specific to psoriasis. To confirm whether the EV miRNA levels depicted a treatment response, we collected blood samples from 10 patients each pre- and post-treatment (Fig. 2D, red arrows). The clinical characteristics of each group are shown in Table 1. Good responders, defined as patients who achieved a 50% reduction in PASI score (PASI50) following treatment, exhibited a significant reduction in levels of miR-625-3p. In contrast, the poor responders (patients who did not achieve a PASI50 after treatment) showed no significant change. No significant changes were observed in miR-4488 for both groups. (Fig. 2F). Taken together, miR-625-3p was the only biomarker that precisely represented psoriasis activity.

### EV miR-625-3p originates from activated keratinocytes

To confirm that miR-625-3p from EVs, rather than complexed circulating miR-625-3p, serves as a novel biomarker of psoriasis severity, we performed mini-size exclusion chromatography (which is also known to isolate EVs effectively) [25] and compared the relative expression of miR-625-3p for each method (Additional file 1: Figure S3). The expression results revealed a significantly strong correlation (Pearson correlation coefficient = 0.8501), indicating that EV miR-625-3p accurately reflected psoriasis severity. Our results also.

**Fig. 3:**
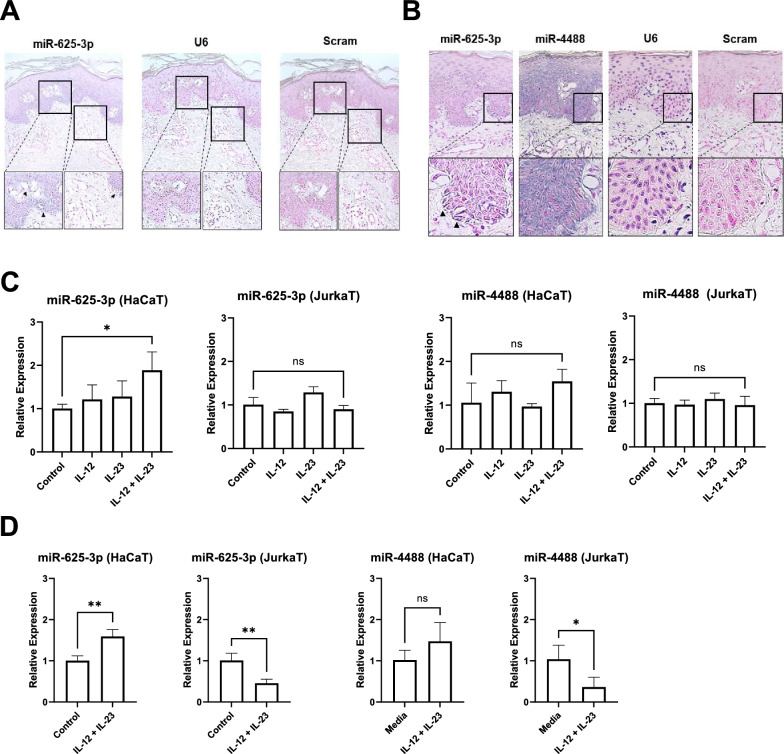
Psoriatic basal keratinocytes as a source of EV miR-625-3p. **A** and **B** The expression of miR-625-3p in the skin detected using ISH in **A** low power field (× 100), and **B** high-power field (× 400). **C** Expression levels of miR-625-3p or miR-4488 measured by qRT-PCR after stimulation with 50 ng/ml IL-12 and/or 50 ng/ml IL-23 for 24 h in collected HaCaT or Jurkat cells. **D** The expression levels of miR-625-3p or miR-4488 in EVs from the cell medium detected by qRT-PCR after stimulation with 50 ng/ml of IL-12 and IL-23 for 24 h. Unpaired Student's t-test was used to compare the two groups. Data are representative of two independent experiments and values are expressed in means ± SEM. *P < 0.05 and **P < 0.01

Since EV miR-625-3p reflected the treatment response and was highly correlated with PASI, we hypothesized that EV miR-625-3p originated from lesional psoriatic skin. To discern the anatomical location of miR-625-3p present in the skin, we performed in situ hybridization (ISH) on the paraffin-embedded skin tissue of patients. Interestingly, miR-625-3p was detected in basal keratinocytes but not in infiltrating immune cells (Fig. 3A). In a high-power field, miR-625-3p was mainly observed in basal keratinocytes' cytoplasm and extracellular matrix (Fig. 3B), in contrast to miR-4488, which exhibited diffuse epidermal staining. We therefore hypothesized that miR-625-3p originates from psoriatic basal keratinocytes. To mimic psoriatic conditions, we stimulated HaCaT cells (human keratinocyte cell line) and Jurkat cells (human T lymphocyte cell line) with IL-12 and IL-23 (both 50 ng/mL), which showed a significant increase in miR-625-3p expression in keratinocytes, not T cells (Fig. 3C). EV miR-625-3p was also increased in the supernatants of stimulated keratinocytes, whereas miR-4488 was not (Fig. 3D). These results suggest that activated keratinocytes are capable of miR-625-3p production and secretion.

### miR-625-3p induces keratinocyte proliferation via IGF-1/Akt signalling interference

**Fig. 4:**
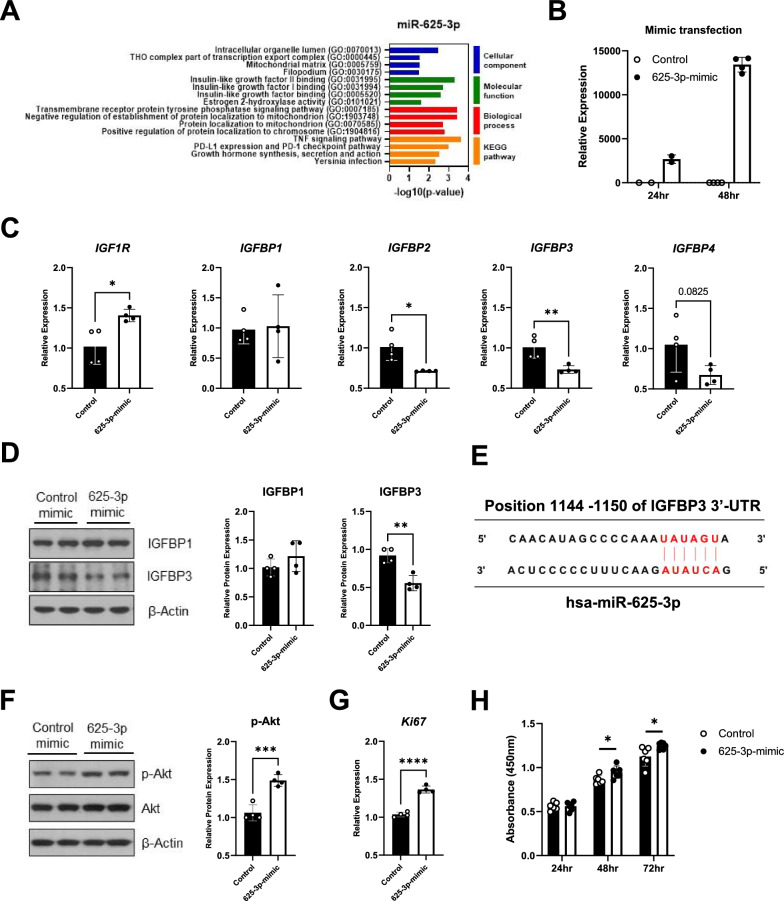
IGF-1 signalling in keratinocytes and its association to miR-625-3p. **A** Prediction of target gene and gene function using three miRNA databases (miRDB/TargetScan/miRTarBase). The overlapping predicted target genes from each database were subjected to GO term and KEGG pathway enrichment analysis. **B** The expression efficiency of miR-625-3p mimic validated using qRT-PCR analysis by HaCaT cells transfection with negative control mimics or miR-625-3p mimics. HaCaT cells were cultured for 48 h after transfection. **C** qRT- PCR results of insulin growth factor -1 receptor (IGF1R) and IGF-binding proteins gene expression. **D** IGFBP1 and IGFBP3 level quantified by western blotting 48 h after transfection. **E** Putative miR-625-3p binding sites in the 3’-UTR of human IGFBP3 mRNA. **F** Western blot analysis of the phospho-Akt protein level. **G** qRT-PCR results of Ki67 gene expression. **H** Cell viability of HaCaT cells transfected with negative control or miR-625-3p-mimic cultured for 24, 48, and 72 h determined using CCK analysis. Data are representative of two independent experiments and values are expressed in means ± SEM. Horizontal lines above bars indicate statistical comparisons with statistical differences between categories. Statistical analyses were performed by the unpaired Student's t-test (**B**–**D**, **F** and **G**) and 2-way ANOVA (**H**). *P < 0.05, **P < 0.01, ****P < 0.001 and ****P < 0.0001

The specific target of miR-625-3p in psoriasis has not yet been identified. Using multiple miRNA databases (Additional file 1: Figure S4), we investigated a possible role in psoriasis pathogenesis, which revealed its possible association with insulin-like growth factor (IGF), particularly with insulin-like growth factor binding (Fig. 4A). Thus, using a miR-625-3p mimic (625-3p mimic), which was successfully transfected to keratinocytes (Fig. 4B), we analyzed the expression levels of insulin-like growth factor binding protein (IGFBP) associated genes and proteins. There was a significant increase in IGF1R and decreases in IGFBP2 and IGFBP3 mRNA expression levels (Fig. 4C). Interestingly, we observed that the protein expression of IGFBP3, and not IGFBP1, was significantly decreased following treatment with the 625-3p mimic (Fig. 4D). Using bioinformatic prediction, we verified the potential miR-625-3p binding site in the human IGFBP3 mRNA (Fig. 4E). These findings suggested the possibility of augmented IGF-1 signalling, which was confirmed by a significant increase in phosphorylated Akt (Fig. 4F). Given that enhanced IGF-1signalling is associated with cell proliferation [26], we evaluated the expression of Ki-67 mRNA, and noted a significant elevation (Fig. 4G). We also observed a significant increase in cell numbers within the miR-625-3p mimic transfected cell population (Fig. 4H).

## Discussion

More than 250 DE miRNAs have been identified in the skin or blood of psoriasis patients [8, 9]. miRNAs are small single-stranded non-coding RNA molecules vulnerable to external stimuli and degradation. However, plasma-derived EV miRNAs are likely more stable, as their encasement within a phospholipid bilayer protects them from degradation in the bloodstream [5, 27]. Thus, we focused on plasma-derived EV miRNAs identified as stable markers of multiple diseases [28, 29], as candidate markers of psoriasis severity.

EV microRNAs have been extensively investigated as potential biomarkers in various diseases, such as autoimmune diseases, cancer, and neurodegenerative diseases [30–32]. They can reflect the secreting cells' characteristics and provide early disease detection opportunities. We identified a single miRNA, miR-625-3p, as a biomarker to determine psoriasis disease activity. miR-625-3p levels were increased in EVs as well as in the skin of patients with psoriasis. While miR-625-3p has been studied previously in cancers such as oral squamous cell carcinoma and malignant melanoma [33–35], studies on EV miR-625-3p are limited. Previous reports showed a decrease in miR-625-3p levels in psoriatic skin compared to non-psoriatic skin, in contrast to our results [36, 37]. This discrepancy might have resulted from the differences in patient characteristics and experimental settings, such as the lack of EV extraction and separation of the epidermis from the dermis [38]. Further evaluation with more patients in a unified experimental setting is necessary for the universal use of our disease severity marker.

To our knowledge, this is the first study to localize the site of miR-625-3p expression in skin. Further, we showed that cytokine-treated KCs could excrete EV miR-625-3p, confirming the origin of the EV miRNA. Considering the proximity of the basal keratinocytes, where EV miR-625-3p is likely secreted, with dermal blood vessels in psoriasis, it seems plausible that these keratinocyte-derived EVs are secreted into the dilated blood vessels and are detectable in circulation.

Recent studies showed that psoriatic keratinocyte-derived EVs can stimulate neutrophils to produce inflammatory cytokines, proliferate resting T cells, and provide immune-stimulatory abilities [38–40]. We present a novel association between IGF-1 signalling and EV miR-625-3p expression in our study. IGF signalling is known for its role in keratinocyte proliferation [26, 41]. Furthermore, IGFBP3 has been suggested as a factor contributing to epidermal hyper-proliferation in psoriasis [42]. Collectively, miR-625-3p may contribute to the pathogenesis of psoriasis through interference with IGF-1 signalling.

Despite the exceptionally high likelihood of disease severity differentiation (based on our AUC value), our study has a few limitations. First, it was conducted in a single center, leading to a relatively small sample size of only one ethnicity. Also, the gender distribution and age range of included patients could potentially be affected by participation bias. Future research needs to confirm the validation of EV miR-625-3p in a larger population with various races and different clinical environments. Second, EV miRNA profiling among samples can be affected by biological and technical variations, including the EV extraction kit’s selectivity, which can lead to inconsistencies between specimens. Third, our study lacked sufficient longitudinal data to reveal the dynamic changes in EV miR-625-3p expression. Future research is imperative to monitor these changes, particularly in the context of long-term therapeutic interventions. Lastly, while we presented a putative target of miR-625-3p, we have not yet elucidated the exact in vivo mechanism of its regulation in psoriasis pathogenesis. We are currently investigating the specific target(s) and their mechanisms in the disease pathogenesis.

## Conclusions

EV miR-625-3p, originating from psoriatic keratinocytes, reflects both the severity of psoriasis and the response to treatment. As there is a current and urgent need for an objective biomarker detectable in the blood that accurately reflects psoriasis disease status, we propose EV miR-625-3p as a potentially useful biomarker for assessing disease activity. This biomarker also holds the potential for being a novel target for psoriasis therapy.

### Supplementary Information

**Additional file 1: Figure S1.** Isolation of plasma EVs by ultracentrifugation (A) NTA demonstrating the size distribution of EVs diluted samples of plasma-derived EVs using ultracentrifugation. (B) Representative TEM image of plasma-derived EVs showing a membrane structure composed of a lipid bilayer (Bar = 200 nm). NTA; nanoparticle tracking analysis; EV; extracellular vesicle; TEM; transmission electron microscope. **Figure S2.** miR-4488 and miR-342-3p from the psoriatic lesional skin show a weak-to-moderate association with PASI and BSA scores. Skin miR-4488 and miR-342-3p levels plotted against PASI and BSA. The significance of the correlation was tested using Spearman's rank correlation test. *P < 0.05. PASI, Psoriasis Area and Severity Index; BSA, body surface area. **Figure S3.** EV miR-625-3p correlates across different isolation methods (A) Representative TEM image of plasma-derived EVs using mini-size exclusion chromatography (Bar = 200 nm). (B) Relative expression levels of EV miR-625-3p isolated using miRCURY exosome Kit showing a strong positive correlation with relative expression of EV miR-625-3p isolated after mini-size exclusion chromatography. Result shown represent combined data of two experiments. The significance of the correlation was tested using the Pearson's correlation test. ****P < 0.0001. **Figure S4.** Venn diagram showing number of predicted gene-targets for miR-625-3p using three different algorithms (miRDB/TargetScan/miRTarBase). The top 100 overlapping genes (6 genes overlapping all three, 94 genes overlapping any two) were chosen for target prediction. **Table S1.** DE microRNA candidates* identified from next-generation sequencing (NGS). **Table S2**. miRNA and mRNAPrimers Used for RT-qPCR.

## Data Availability

miRNA sequencing data will be deposited in the Gene Expression Omnibus database (http://www.ncbi.nlm.nih.gov/geo/) under the accession number GSE215124. All other data from this study are available from the corresponding author upon reasonable request.

## Abbreviations

AP: Alkaline phosphatase
AUC: Area under the curve
BD: Behçet’s disease
BSA: Body surface area
DE: Differentially expressed
EV: Extracellular vesicle
HRP: Horseradish peroxidase
IGF: Insulin-like growth factor
IL: Interleukin
miRNAs: MicroRNAs
PASI: Psoriasis Area and Severity Index
qRT-PCR: Real-time quantitative reverse transcription-PCR
SDS: Sodium dodecyl sulfate
SEM: Standard error of mean
SSC: Saline-sodium citrate
