# Supplementary material for: Keratinocyte-derived circulating microRNAs in extracellular vesicles: a novel biomarker of psoriasis severity and potential therapeutic target
Source: J Transl Med. 2024 Mar 4;22:235. doi: 10.1186/s12967-024-05030-z (PMC10910723; doi:10.1186/s12967-024-05030-z)
Supplement: Supplementary file 1 — Additional file 1: Figure S1. Isolation of plasma EVs by ultracentrifugation (A) NTA demonstrating the size distribution of EVs diluted samples of plasma-derived EVs using ultracentrifugation. (B) Representative TEM image of plasma-derived EVs showing a membrane structure composed of a lipid bilayer (Bar = 200 nm). NTA; nanoparticle tracking analysis; EV; extracellular vesicle; TEM; transmission electron microscope. Figure S2. miR-4488 and miR-342-3p from the psoriatic lesional skin show a weak-to-moderate association with PASI and BSA scores. Skin miR-4488 and miR-342-3p levels plotted against PASI and BSA. The significance of the correlation was tested using Spearman's rank correlation test. *P < 0.05. PASI, Psoriasis Area and Severity Index; BSA, body surface area. Figure S3. EV miR-625-3p correlates across different isolation methods (A) Representative TEM image of plasma-derived EVs using mini-size exclusion chromatography (Bar = 200 nm). (B) Relative expression levels of EV miR-625-3p isolated using miRCURY exosome Kit showing a strong positive correlation with relative expression of EV miR-625-3p isolated after mini-size exclusion chromatography. Result shown represent combined data of two experiments. The significance of the correlation was tested using the Pearson's correlation test. ****P < 0.0001. Figure S4. Venn diagram showing number of predicted gene-targets for miR-625-3p using three different algorithms (miRDB/TargetScan/miRTarBase). The top 100 overlapping genes (6 genes overlapping all three, 94 genes overlapping any two) were chosen for target prediction. Table S1. DE microRNA candidates* identified from next-generation sequencing (NGS). Table S2. miRNA and mRNAPrimers Used for RT-qPCR. [file 12967_2024_5030_MOESM1_ESM.docx]

**Additional Materials and methods**

**Transmission electron microscopy (TEM)**

Freshly isolated EVs were deposited onto EM grids for 10 min. The vesicle-coated grids were washed three times with PBS and fixed. After washing with distilled water (DW), the grids were stained with 2% uranyl acetate for 15 minutes, and dried for 30 minutes. TEM was performed using a Field Emission Scanning Electron Microscope (Sigma 500, Carl Zeiss, Oberkochen, Germany) at the Three-Dimensional Immune System Imaging Core Facility of Ajou University.

**Nanoparticle tracking analysis (NTA)**

Isolated extracellular vesicles (EV) were analyzed by ExoCope mono (ExosomePLUS, Suwon, Republic of Korea) to measure the size and number of EV particles. Samples are diluted with filtered PBS to achieve an appropriate event per frame (~1000 dilutions for below 150 events/frame). 144 frames are recorded at each position, and repeated at 10 different sub-volume positions. The data is analysed with ExoCope HQ ver.1300. The smallest target diameter is 50 nm, and the minimum particle distance is 2 times of mean squared distance of a 50 nm particle.

**miRNA sequencing**

Before library preparation, all RNA samples underwent quality control processes by the Theragen Bio (Suwon, Republic of Korea)5. RNA samples with RNA integrity number (RIN) values of 6 or higher were deemed suitable for further analysis. The libraries were prepared for 50 bp single-end sequencing using the NEXTflex Small RNA-Seq Kit v3 (Bioo Scientific Corp.). Namely, smallRNA molecules were isolated from 1 μg of total RNA via the adapter ligation. The isolated smallRNAs were synthesised as single-stranded cDNAs through RT (Reverse transcription) priming. When this was applied as a template for second-strand synthesis, double-stranded cDNA was prepared by PCR. The fragments around 150 bp were extracted for sequencing through size selection by gel electrophoresis. The quality of these cDNA libraries was evaluated with the Agilent 2100 BioAnalyzer (Agilent, CA, USA) followed by quantification with the KAPA library quantification kit (Kapa Biosystems, MA, USA) according to the manufacturer’s protocol. Following cluster amplification of the denatured templates, sequencing was progressed as paired-end (150 bp) using the Illumina NovaSeq6000 S4 sequencing platform (Illumina, CA, USA).

**miRNA NGS data processing**

The miRNA expression level was measured with mirdeep2^15^ using the gene annotation database of the species along with hairpin and mature miRNA sequence information which can be extracted from miRbase.^16^ For DEmiRNA analysis, miRNA level count data were generated using mirdeep2.^15^ Based on the calculated read count data, DEmiRNAs were identified using the TCC R package^17^, where this package applies robust normalisation strategies to compare tag count data. Normalisation factors were calculated using the iterative DEGES/edgeR method. This method was selected for its ability to effectively account for variability in sequencing depth and suitability for our study, as it accommodates the small sample sizes we worked with. Q-value was calculated based on the p-value using the p.adjust function of R package with default parameter settings. DEmiRNAs were identified based on the q-value threshold less than 0.05 for correcting errors caused by multiple-testing.^18^

**miRNA real-time qPCR**

cDNA was produced using miRCURY LNA miRNA PCR Assays (Qiagen) with a miRCURY LNA RT Kit (Qiagen), according to the manufacturer’s instructions. qRT-PCR was performed using the QuantStudio 3 Real-Time PCR System (Applied Biosystems, Foster City, CA, USA). The miRNA expression values obtained by RT-qPCR were normalised to those of miR-103-3p.^19^ The relative standard curve method (2^−ΔΔCT^) was used to quantitatively analyse the results. Primers were purchased from Qiagen. For primer sequences, please see Table E1 in the Online Repository.

**In situ hybridization**

Formalin-fixed paraffin-embedded sections were cut (6-μm thick) and mounted on slides. The miRCURY LNA miRNA Detection Probe (Qiagen) was used for miRNA in situ hybridization (ISH), and the experiment was performed according to the manufacturer’s protocols. Briefly, sections of the lesional specimens of the patients were deparaffinised and treated with proteinase K for 10 min at 37°C. The hybridisation mix was prepared by adding the LNA detection probes for hsa-miR-625-3p and hsa-miR-4488 (Qiagen, both 5′-DIG– and 3′-DIG–labelled) to the miRNA ISH buffer (Qiagen). This mix was then applied to the incubated slides and was placed in the hybridiser at temperatures ranging from 55°C to 60°C. The slides were then washed with 5× Saline-Sodium Citrate (SSC), 1× SSC, and 0.2× SSC buffer, followed by incubation in 0.2× SSC buffer for 1 h at 60°C. After stringent washes, the probes were detected by incubating the sections with alkaline phosphatase (AP)-conjugated anti-DIG Ab for 1 h at room temperature. AP substrate was added to the slides for 2 h at 30°C in a humidifying chamber. The AP reaction was stopped using KTBT buffer according to the manufacturer’s instructions. The slides were counterstained, rinsed, and dehydrated before analysis by light microscopy.

**Treatment of IL-12 and IL-23 on cell lines**

Immortalised human keratinocyte (HaCaT) and T (JurkaT) cells (kindly obtained from Dr. Tae-Gyun Kim, Yonsei University, South Korea, and Dr. Hee Young Kang, Ajou University, South Korea, respectively) were cultured. The cell lines tested negative for mycoplasma and were grown in Roswell Park Memorial Institute (RPMI) medium 1640 with L-glutamine supplemented with 10% heat-inactivated foetal calf serum. HaCaT cells and Jurkat cells were maintained at concentrations of 4 × 10^4^ cells and 5 × 10^5^ cells, respectively, in each medium in a 12-well plate, and interleukin (IL)-12 and/or IL-23 were added to each well plate. The cells were harvested after 24 h. Total RNA from HaCaT and Jurkat cells was isolated using the miRNeasy Micro Kit (Qiagen) according to the manufacturer’s protocols.

**miRNA target prediction**

miRNA targets were predicted separately using an online database for miRNA target prediction and functional annotations: miRDB^20^, TargetScan^21^, and miRTarBase^22^. From miRDB, TargetScan 8.0, and mirRTarBase 9.0, 294, 2906, and 33 targets, respectively, were predicted to be targets of miR-625-3p. We chose the top 100 overlapping genes (6 genes overlapping all three, 94 genes overlapping any two) and used the Enrichr tool^23^ to obtain enrichment scores (P-values) for KEGG pathways and GO terms, using HGNC names as input. Only the top four (lowest P-values) for each category are reported.

**Transfection of miRNA mimics**

The miRNA mimics (negative control mimic and miR-625-3p mimic) were designed and constructed using Thermo Fisher Scientific. HaCaT cells were transfected using Lipofectamine® RNAiMAX reagent (Thermo Fisher Scientific, Inc.). These cells (2 x 10^5^ cells/6 wells) were then seeded into growth plates and incubated for 24 h, after which they were transfected with a 50 pmol miRNA mimic, following the manufacturer’s protocols. After transfection, the cells were harvested for subsequent experiments.

**Western blotting**

Transfected HaCaT cells were lysed with RIPA buffer (Thermo Fisher Scientific) containing a protease inhibitor cocktail (Merck) to extract cellular proteins. Equivalent amounts of protein (20 μg) in sodium dodecyl sulfate (SDS) sample buffer were separated by 10% SDS polyacrylamide gel electrophoresis and transferred to polyvinylidene difluoride (PVDF) membranes (Millipore). After blocking with 5% skim milk for 30 min, the membranes were incubated with primary antibodies against p-Akt (1:1000, Cell Signaling; #9271), Akt (1:1000, Cell Signaling; #9272), or β-actin (1:10000, Bethyl; A300-491A) at 4°C. The sections were then incubated with secondary horseradish peroxidase (HRP)-conjugated anti-rabbit IgG antibodies (Enzo) at room temperature for 2 h. Immunoreactive bands were detected using the Enhanced Chemiluminescence System (Pierce ECL Western Blotting Substrate; Thermo Fisher Scientific). The densitometric quantification of the p-Akt protein was performed using ImageJ with β-actin as a loading control.

**Cell counting kit (CCK) assay**

HaCaT cells were seeded in 96-well plates. Cell viability was measured using the CCK cell viability assay kit (Dongin Biotech, Seoul, Korea), in accordance with the manufacturer’s protocol. Briefly, 10 µL of CCK reagent was added to each well at the indicated time points (24, 48, or 72 h) after transfection and incubated for 2 h at 37°C. Absorbance at 450 nm was measured using an EPOCH microplate reader (BioTek Instruments, Winooski, VT, USA).

**Supplementary figures**


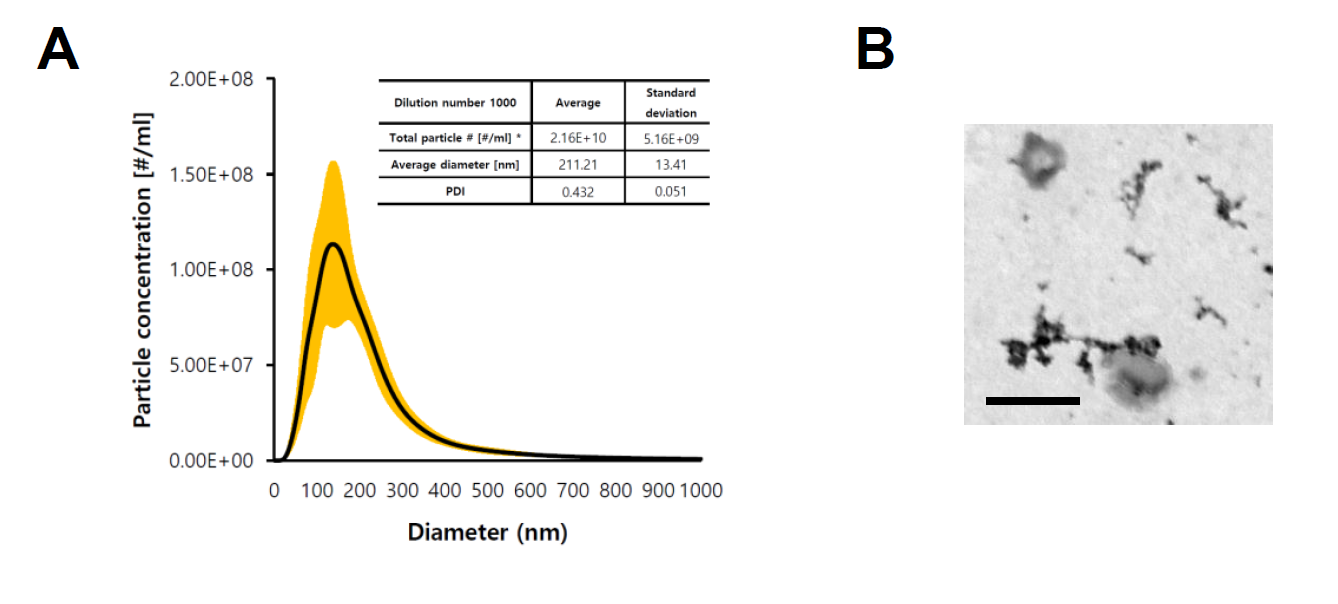


**Fig S1.** Isolation of plasma EVs by ultracentrifugation (A) NTA demonstrating the size distribution of EVs diluted samples of plasma-derived EVs using ultracentrifugation. (B) Representative TEM image of plasma-derived EVs showing a membrane structure composed of a lipid bilayer (Bar = 200 nm). NTA; nanoparticle tracking analysis; EV; extracellular vesicle; TEM; transmission electron microscope.


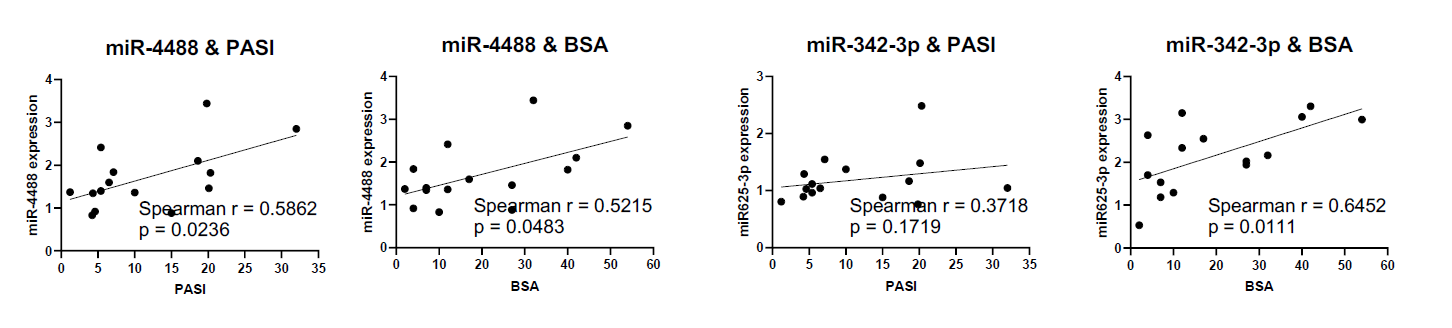
 **Fig S2.** miR-4488 and miR-342-3p from the psoriatic lesional skin show a weak-to-moderate association with PASI and BSA scores. Skin miR-4488 and miR-342-3p levels plotted against PASI and BSA. The significance of the correlation was tested using Spearman's rank correlation test. *P < 0.05. PASI, Psoriasis Area and Severity Index; BSA, body surface area.


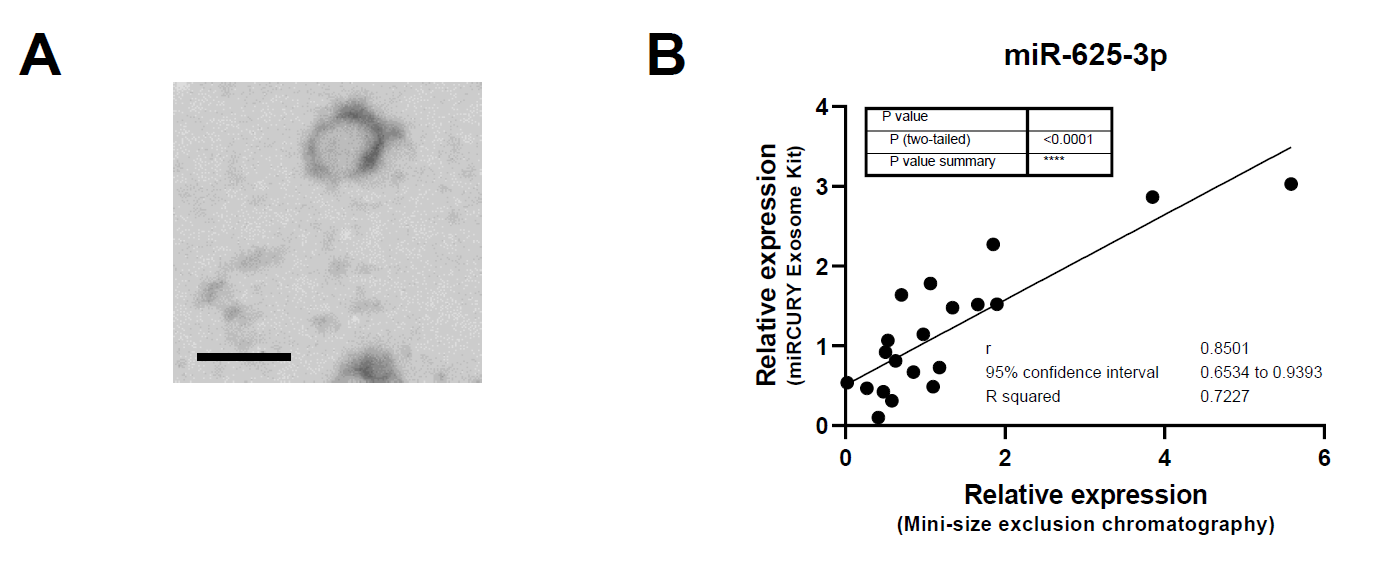
 **Fig S3.** EV miR-625-3p correlates across different isolation methods (A) Representative TEM image of plasma-derived EVs using mini-size exclusion chromatography (Bar = 200 nm). (B) Relative expression levels of EV miR-625-3p isolated using miRCURY exosome Kit showing a strong positive correlation with relative expression of EV miR-625-3p isolated after mini-size exclusion chromatography. Result shown represent combined data of two experiments. The significance of the correlation was tested using the Pearson's correlation test. ****P < 0.0001


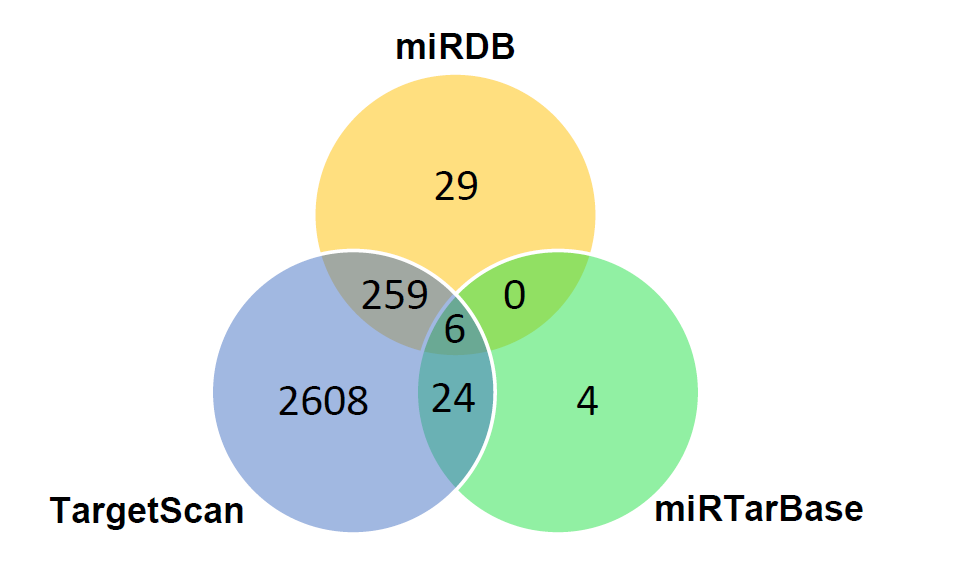


**Fig S4.** Venn diagram showing number of predicted gene-targets for miR-625-3p using three different algorithms (miRDB/TargetScan/miRTarBase). The top 100 overlapping genes (6 genes overlapping all three, 94 genes overlapping any two) were chosen for target prediction.

**Additional Table 1. DE microRNA candidates* identified from next-generation sequencing (NGS)**

| microRNA | Fold change | *P*-value |
| --- | --- | --- |
| miR-625-3p | 2.00 | 0.006472 |
| miR-5698 | -8.66 | 0.008873 |
| miR-342-3p | 2.40 | 0.010319 |
| miR-1268a | -4.54 | 0.016039 |
| miR-423-3p | 1.54 | 0.018738 |
| miR-4488 | 10.43 | 0.024191 |
| miR-328-3p | 1.33 | 0.025667 |
| miR-6514-5p | -7.89 | 0.026176 |
| miR-1255b-5p | -8.50 | 0.032926 |
| miR-150-5p | 1.95 | 0.033285 |
| miR-4466 | 7.97 | 0.037048 |
| miR-1468-5p | -7.60 | 0.037296 |
| miR-1228-3p | -6.58 | 0.03798 |
| miR-1268b | -4.43 | 0.038019 |
| miR-27a-3p | 1.61 | 0.039127 |
| miR-656-3p | -3.89 | 0.041276 |
| miR-7106-3p | -7.30 | 0.042793 |
| miR-323a-5p | -7.66 | 0.04774 |
| miR-150-3p | 2.53 | 0.049293 |

*These miRNAs concurrently meet the conditions of |log2 fold change| ≥ 2 and P ≤ 0.05.

**Supplementary Table 2.** miRNA and mRNA Primers Used for RT-qPCR

| **miRNA Primers Used for Reverse Transcription (the Stem-Loop Method)** | | | |
| --- | --- | --- | --- |
| Gene name | |  | |
| hsa-miR-4488 | | 5′-AGG GGG CGG GCT CCG GCG-3′ | |
| hsa-miR-342-3p | | 5′-TCT CAC ACA GAA ATC GCA CCC GT-3′ | |
| hsa-miR-625-3p | | 5′-GAC TAT AGA ACT TTC CCC CTC A-3′ | |
| hsa-miR-1255b-5p | | 5′-CGG ATG AGC AAA GAA AGT GGT T-3′ | |
| hsa-miR-323a-5p | | 5′-AGG TGG TCC GTG GCG CGT TCGC-3′ | |
| hsa-miR-5698 | | 5′-TGG GGG AGT GCA GTG ATT GTGG-3′ | |
| **RNA Primers Used for qRT-PCR** | | | |
| Gene name | Forward | | Reverse |
| IGF1R | 5′-GCA CCA TCT TCA AGG GCA ATT TG-3′ | | 5′-AGG AAG GAC AAG GAG ACC-AAG G-3′ |
| IGFBP1 | 5′-AGG CTC TCC ATG TCA CCA AC-3′ | | 5′-CCT GTG CCT TGG CTA AAC TC-3′ |
| IGFBP2 | 5′-CCT CTA CTC CCT GCA CAT CC-3′ | | 5′-TGC CCG TTC AGA GAC ATC TT-3′ |
| IGFBP3 | 5′-TGT GGC CAT GAC TGA GGA AA-3′ | | 5′-TGC CAG ACC TTC TTG GGT TT-3′ |
| IGFBP4 | 5′-ACT CTG CTG GTG CGT CTA CC-3′ | | 5′-TAT CTG GCA GTT GGG GTC TC-3′ |
| GAPDH | 5′-GAA CGG GAA GCT CAC TGG-3′ | | 5′-GCC TGC TTC ACC ACC TTC T-3′ |
